# Fuzzy jump wavelet neural network based on rule induction for dynamic nonlinear system identification with real data applications

**DOI:** 10.1371/journal.pone.0224075

**Published:** 2019-12-09

**Authors:** Mohsen Kharazihai Isfahani, Maryam Zekri, Hamid Reza Marateb, Miguel Angel Mañanas

**Affiliations:** 1 Department of Electrical and Computer Engineering, Isfahan University of Technology, Isfahan, Iran; 2 Biomedical Engineering Department, Engineering Faculty, University of Isfahan, Isfahan, Iran; 3 Biomedical Engineering Research Centre (CREB), Automatic Control Department (ESAII) Universitat Politècnica de Catalunya-Barcelona Tech (UPC), Barcelona, Spain; 4 Biomedical Research Networking Center in Bioengineering, Biomaterials, and Nanomedicine (CIBER-BBN), Spain; Vietnam National University, VIETNAM

## Abstract

**Aim:**

Fuzzy wavelet neural network (FWNN) has proven to be a promising strategy in the identification of nonlinear systems. The network considers both global and local properties, deals with imprecision present in sensory data, leading to desired precisions. In this paper, we proposed a new FWNN model nominated “Fuzzy Jump Wavelet Neural Network” (FJWNN) for identifying dynamic nonlinear-linear systems, especially in practical applications.

**Methods:**

The proposed FJWNN is a fuzzy neural network model of the Takagi-Sugeno-Kang type whose consequent part of fuzzy rules is a linear combination of input regressors and dominant wavelet neurons as a sub-jump wavelet neural network. Each fuzzy rule can locally model both linear and nonlinear properties of a system. The linear relationship between the inputs and the output is learned by neurons with linear activation functions, whereas the nonlinear relationship is locally modeled by wavelet neurons. Orthogonal least square (OLS) method and genetic algorithm (GA) are respectively used to purify the wavelets for each sub-JWNN. In this paper, fuzzy rule induction improves the structure of the proposed model leading to less fuzzy rules, inputs of each fuzzy rule and model parameters. The real-world gas furnace and the real electromyographic (EMG) signal modeling problem are employed in our study. In the same vein, piecewise single variable function approximation, nonlinear dynamic system modeling, and Mackey–Glass time series prediction, ratify this method superiority. The proposed FJWNN model is compared with the state-of-the-art models based on some performance indices such as RMSE, RRSE, Rel ERR%, and VAF%.

**Results:**

The proposed FJWNN model yielded the following results: RRSE (mean±std) of 10e-5±6e-5 for piecewise single-variable function approximation, RMSE (mean±std) of 2.6–4±2.6e-4 for the first nonlinear dynamic system modelling, RRSE (mean±std) of 1.59e-3±0.42e-3 for Mackey–Glass time series prediction, RMSE of 0.3421 for gas furnace modelling and VAF% (mean±std) of 98.24±0.71 for the EMG modelling of all trial signals, indicating a significant enhancement over previous methods.

**Conclusions:**

The FJWNN demonstrated promising accuracy and generalization while moderating network complexity. This improvement is due to applying main useful wavelets in combination with linear regressors and using fuzzy rule induction. Compared to the state-of-the-art models, the proposed FJWNN yielded better performance and, therefore, can be considered a novel tool for nonlinear system identification.

## Introduction

System identification is a challenging work in the many fields of engineering, which is concerned with achieving the model of dynamic nonlinear or linear systems based on the input and output observations, especially from experimental data with prior knowledge or inadequate information [1]. In recent years, many studies have been conducted using the combination of computational intelligence methods for nonlinear dynamic system modeling, function approximation, and time-series prediction [2–9].

Neural networks (NN) and fuzzy systems as computational intelligence methods are suitable tools for modeling expert knowledge and dealing with uncertain nonlinear processes or time series [8]. Incorporating NNs, wavelets, and fuzzy inference systems offer sophisticated solutions, named fuzzy wavelet neural networks (FWNN) [3–9]. The FWNN employs the learning ability of neural networks, time-frequency localization property of wavelets, and approximate reasoning characteristics of fuzzy systems to present a practical model handling uncertainty and disturbances in real data for complex hybrid nonlinear-linear problems. Hence, FWNNs require lower training time and have fewer rules and higher efficiency than fuzzy systems or neural networks [10].

FWNN model is a traditional Takagi-Sugeno-Kang neuro-fuzzy system in which the consequent part of fuzzy rules is replaced by a wavelet neural network. The antecedent part of each fuzzy rule in the FWNN model divides the input space into local fuzzy regions, and its consequent part corresponds to a sub-wavelet neural network [11].

In [3], the nonlinear autoregressive moving average with exogenous inputs was identified by a dynamic time-delay fuzzy wavelet neural network. In [12], a fuzzy wavelet neural network was proposed for nonlinear function approximation. In that network, a sub-wavelet neural network consisting of single-scaling wavelets was introduced for the consequent part of each fuzzy rule. The adaptive type of the network was developed in [8] as a solution for controlling nonlinear affine systems. Moreover, to identify and regulate nonlinear systems, the summation form of multidimensional wavelet functions constituted the fuzzy rule consequent part of the FWNN [4]. In [7], FWNN model membership functions in the antecedent part were wavelets similar to the activation functions in the consequent part of fuzzy rules.

Despite various FWNN model advantageous, they cannot correctly deal with systems with both linear and nonlinear dynamics. Also, inappropriate regulation of wavelet parameters reduces the generalizability of the model [13]. Also as another challenge of the FWNN, it is not a simple work to extract effective fuzzy rules [14]. The mentioned challenges are significant problems, especially in practical applications. For example, FWNN application in blood glucose concentration prediction in [15] has not worked so well in comparison with other types of neural networks. On the other hand, wavelet-based models for dynamical systems lead to a large number of neurons and time delay lines. This issue increases the complexity of the network [16].

Therefore, methods such as C-means clustering are used to enhance the FWNN structure [14, 17]. For example, in Fuzzy wavelet polynomial neural networks described in [17], the dominant selected wavelets were classified, and then each class was placed in the then-part of a fuzzy rule. In that study, C-means clustering was implemented in the antecedent of the fuzzy rules instead of Gaussian membership functions. In a similar example in [14], a self-adapted fuzzy C-means clustering was used to determine the number of fuzzy rules of the FWNN model. While promoting the structure of fuzzy rules by applying C-means clustering is sensitive to noise, not comprehensive and only affects the number of fuzzy rules.

This paper presents a new FWNN model named as fuzzy jump wavelet neural network (FJWNN) for identification dynamic nonlinear-linear systems. The proposed FJWNN provides modeling by using input regressors and their wavelet transform in the consequent part of each fuzzy rule. Consequently, each fuzzy rule can locally model both linear and nonlinear properties of a system. The linear relationship between the inputs and the output is learned by neurons with linear activation functions whereas nonlinear relationship is locally modeled by wavelet neurons. The OLS and GA methods are respectively applied to extract dominant wavelets exerting the most significant effect on the output. In selecting dominant wavelets, it is supposed that wavelet neurons are along with the linear combination of input regressors. Then, to optimize the proposed FJWNN structure including the number of fuzzy rules, effective inputs of each fuzzy rule, and model parameters, fuzzy rule induction is used. The performance of FJWNN in experimental applications is illustrated by applying the real-world Box-Jenkins gas furnace system and the electromyographic (EMG) signal modeling problem. Also, well-known benchmarks in function approximation, identification of dynamic nonlinear systems and time series prediction are studied to identify the ability of the proposed FJWNN model, in comparison with the state-of-the-art models. The main features of the FJWNN model proposed in this work are:

1. In our approach, it is possible to handle real data problems of large dimensions because the effective procure of choosing wavelets used in OLS method is not very sensitive to the input dimension. It is worth noting that the role of GA combined with OLS is on selecting the most influential wavelets. While in most reported neuro-fuzzy models such as [9, 18, 19], GA is determined to train unknown parameters.
2. The proposed FJWNN utilizes input regressors, and their wavelet transforms in the consequent part of each fuzzy rule. Consequently, wavelets with different scale values combined with input regressors under fuzzy rules are fully used to capture different global or local behaviors of dynamic nonlinear-linear systems.
3. By applying fuzzy rule induction [20], it is possible to assign a weight to each fuzzy rule, which determines the importance of each fuzzy rule. Consequently, when defining a proper threshold, insignificant rule is removed and it leads to a more straightforward structure. Also, in comparison with [14, 17], in our research, fuzzy rule induction prunes unnecessary inputs from each of the fuzzy rules.

This paper is organized as follows. The materials and methods section introduces benchmarks and presents the FJWNN model. FJWNN modeling results and their interpretation in comparison with the state-of-the-art models are provided in the results and discussion section. The final section provides some concluding remarks.

## Materials and methods

### Materials

The study materials consist of simulated benchmark examples and experimental problems. Function approximation, identification of dynamic nonlinear systems and time series prediction as machine learning problems and Box-Jenkins gas furnace system and the EMG signal modeling problem as input-output measurements of real datasets are taken into account in this study to identify the ability of the proposed FJWNN model, compared to the state-of-the-art models.

### Simulated datasets

Multiple simulated benchmarks including a piecewise single variable function, five nonlinear dynamic plants with various nonlinear structures, and the chaotic Mackey Glass time series (with different signal to noise ratio (SNR) and various chaotic degrees) are considered to verify the effectiveness of the proposed FJWNN model. Comparing simulation results in the following examples, the conditions are considered similar to their corresponding references.

#### Example 1—Function approximation

The piecewise single variable function, which has been studied in the literature frequently [5, 6, 12], is described as follows:

$$f(x)=\{\begin{array}{c} -2.186\;x-12.864\;,\\ 4.246\;x\;,\\ 10\;e^{-\;0.05\;x\;-\;0.5}\text{sin}\left[\left(0.03\;x\;+0.7\right)\;x\right]\;, \end{array}\begin{array}{c} -10\leq x<-2\\ -2\leq x<0\\ 0\leq x\leq 10 \end{array}$$ (1)

The training data is composed of 200 input-output pairs uniformly sampled in the region [–10, 10], as mentioned in [5, 6, 12].

#### Example 2—Dynamic nonlinear system identification

Illustrating the ability of proposed approach in identification of dynamic nonlinear systems, five systems are considered in the following. In these examples, multiple different nonlinearity structures are used.

This example (Example 2–1) is a nonlinear dynamic system defined, which has been studied in the literature frequently [4, 11, 21–23], as follows:

$$\begin{array}{l} y(k)\;=\;0.72\;\;y(k-1)\;\;+\;\;0.025\;\;y(k-2)\;\;u(k-2)\;\\ \;+\;\;0.01\;\;u{\left(k-3\right)}^{2}\;+\;\;0.2\;\;u(k-4) \end{array}$$ (2)

The input training signal is an independent and identically distributed uniform sequence over [–2,2] for about half of the 900 time steps and a sinusoid given by 1.05sin(πk⁄45) for the remaining time. Also, the following input signal is used as the test input signal:

$$\;u(k)=\{\begin{array}{c} \begin{array}{cc} \text{sin}\;(\pi \;k/25) & , \end{array}\\ \begin{array}{cc} +\;1.0 & , \end{array}\\ \begin{array}{cc} -\;1.0 & , \end{array}\\ \begin{array}{cc} \begin{array}{l} 0.3\;\text{sin}\;(\pi \;k/25)\;+\;0.1\;\text{sin}\;(\pi \;k/32)\;+\;\\ 0.6\;\text{sin}\;(\pi \;k/10) \end{array} & , \end{array} \end{array}\;\;\;\;\begin{array}{c} k\leq 250\\ 250<k\leq 500\\ 500<k\leq 750\\ 750<k\leq 1000 \end{array}$$ (3)

In the following plants [24–27], different structures including nonlinearity in relative to input and its delays, output delays or both of them are considered (Examples 2–2, 2–3, 2–4 and 2–5) respectively

$$\begin{array}{l} y(k)=0.3y(k-1)+0.6y(k-2)+0.6\text{sin}(\pi u(k))+\\ \;0.3\text{sin}(3\pi u(k))+0.1\text{sin}(5\pi u(k)) \end{array}$$ (4)

$$\;y(k)=\frac{y\left(k-1\right)y\left(k-2\right)\left[y\left(k-1\right)+0.5\right]\left[y\left(k-2\right)-1\right]}{1+y^{2}\left(k-1\right)+y^{2}\left(k-2\right)}+u(k)$$ (5)

$$\begin{array}{l} \;y(k)=\frac{y(k-1)\left[y(k-1)+0.3\right]}{1+y^{2}(k-1)}+\\ \;\;\;\;\;\;\;\;\;\;\;\;\;\;u(k-1)\left[u(k-1)+0.8\right]\left[u(k-1)-0.5\right] \end{array}$$ (6)

$$\;y(k)=\frac{y(k-1)y(k-2)y(k-3)u(k-2)[y(k-3)-1]}{1+y^{2}(k-2)+y^{2}(k-3)}$$ (7)

For all these four plants, training input signal was taken from a 1000 time step uniformly distributed random signal over the interval [-1,+1] and test input signal is a 600 time step sinusoidal signal as given by

$$\;u(k)=\{\begin{array}{cc} \begin{array}{c} \text{sin}\left(\frac{2\pi k}{250}\right),\\ 0.8\text{sin}\left(\frac{2\pi k}{250}\right)+0.2\text{sin}\left(\frac{2\pi k}{25}\right), \end{array} & \begin{array}{c} 0\leq k\leq 250\\ \begin{array}{l} 250\leq k\leq 600 \end{array} \end{array} \end{array}$$ (8)

#### Example 3—Predicting chaotic time series

This simulation example is the Mackey-Glass time series, which is considered as a prediction problem used in [28–33] as a benchmark example. The Mackey–Glass system was introduced as a white blood cell production model [34]. This time series is obtained from the following delay differential equation:

$$d\;x(t)\;/\;dt\;\;=\;[\;0.2\;\;x\;(t-\tau )\;]\;/\;[\;1\;\;+\;\;x^{10}(t\;-\;\tau )\;]\;-\;\;0.1\;\;x\;(t)$$ (9)

with x(0) = 1.2, τ = 17, and x(t) = 0 for t < 0 and regardless of the noise. 1200 input-output data points were generated while data pairs with t = 124 to 1123 were chosen for system identification. The first 500 points were used for training, and the remaining data were used for testing. Similar to [28–33], the following input regressors were selected to identify the output x(t).

[x(t−6)x(t−12)x(t−18)x(t−24)] (10)

In following steps, the Mackey-Glass time series for other different values of tau (τ = 13, τ = 30 and τ = 100) and also corrupted data by uniformly-distributed stationary additive noise (SNR = 0 dB, SNR = 10 dB and SNR = 20 dB) are considered.

### The real dataset

#### Example 4—Real-world Box-Jenkins gas furnace system

The Box-Jenkins system [35] is a complicated nonlinear system. The benchmark dataset consists of 296 input-output measurements of a real-world gas furnace process. The measurements as a time series data include the gas flow rate u(k) and the CO2 concentration y(k). In [1], y (k-1), y(k-2), y(k-3), u(k), u(k-1), u(k-2) are chosen as the model inputs. The number of 296 samples is equally divided into two parts which the first part is used for training, and the second is used for testing.

#### Example 5—EMG signal modeling problem

In this example, the EMG signal modeling is considered. This application is a necessary step in the load-sharing problem, a suitable solution for movement analysis, described in [36–38]. This problem estimates the individual mechanical contribution of the muscles acting on the same joint based on their electromyography (EMG) and the total torque [39]. Data related to the physiological processes during muscle contraction is provided in surface electromyography (sEMG) envelops. Since the force produced by a particular muscle cannot be measured, it is usually estimated from sEMG envelops [40, 41]. The problem is the estimation of the torque exerted on a joint using the EMG signals of the contracted muscles.

In this study, the dataset provided in [36] was used. The inclusion criteria of the participants were no sign of the previous neuromuscular disorders. Also, the sampling method was convenience non-random sampling. Five healthy males (age: 21.3 ± 2.8 years; height 174.3 ± 2.6 cm; body mass 71.0 ± 3.4 kg) performed three series of flexion-extension force ramps. Each series lasted 25 s, and contrariwise (with n = 30, 50, 70) involved four isometric extension (e) and flexion (f) ramps from n% eMVC to n% fMVC. Data used in this study was obtained from a previous study [36], where written informed consent in accordance with the declaration of Helsinki was confirmed by each participant and the experimental protocol was approved by the ethical committee of the Politecnico di Torino.

Isometric voluntary flexions (extensions with elbows flexed at 90°) stored sEMG signals from the Biceps Brachii (BB), Brachioradialis (BR), and lateral and medial heads of the Triceps Brachii (TBL and TBM). The signals were acquired from BR, TBL, and TBM with three linear arrays of 8 electrodes (5-mm inter-electrode intervals). Moreover, an isometric brace, used for limb fixation, was used for measuring the torque signal. Then, the signal was amplified using Force Amplifier MISO-II (LISiN, Politecnico di Torino, Italy) and then sampled at 2048 Hz.

Single differential (SD) and double differential (DD) signals were calculated along with Fiber direction. A non-causal digital low-pass filter (1 Hz, 4^th^-order Butterworth filter) derived the envelope of sEMG signals from rectified signals. The spatial mean of the recorded signals of each muscle was considered the global envelope for the muscle. The mean number of data samples for each experiment was 796. The data was provided in Figshare (https://figshare.com/s/6c772ef829faf53240c0).

## The proposed fuzzy jump neural network model

**Fig 1:**
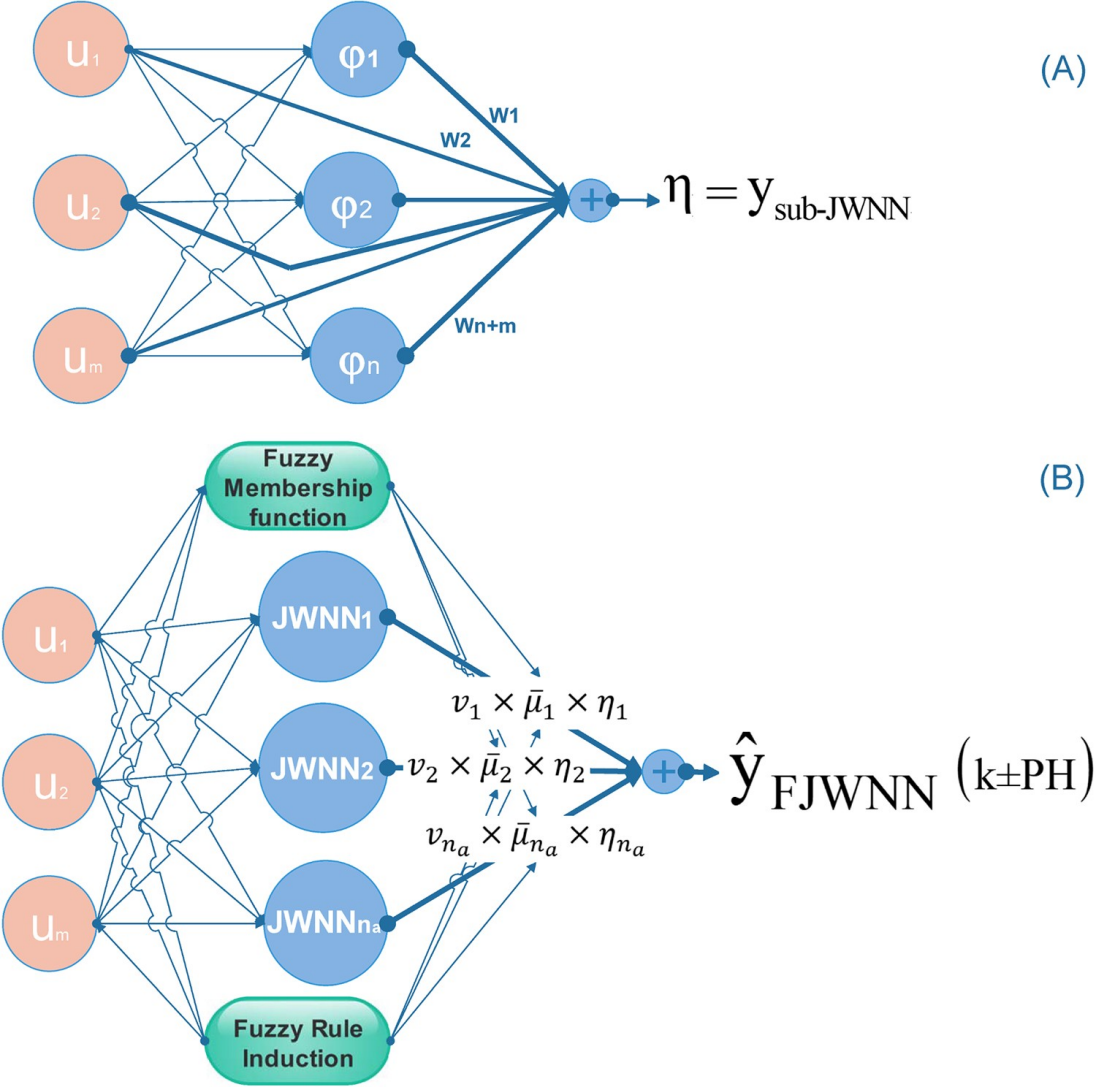
(A) The sub-JWNN structure and (B) the proposed FJWNN model structure. In this figure, where *u*_*1*_, *u*_*2*_, *…*, *u*_*m*_ are inputs, *φ*_*1*_, *φ*_*2*_, *…*, *φ*_*n*_ are the selected dominant wavelet neurons, *W*_*1*_,*W*_*2*_, *…*,*W*_*n+m*_ are weights of the sub-JWNN output layer, *JWNN1*, *JWNN2*, *…*, *JWNN*_*na*_ are n_a_ sub-JWNN made from n dominant selected wavelets, *η*_*1*_,*η*_*2*_, *…*,*η*_*na*_ are outputs of the *n*_*a*_ sub-JWNN, *v*_*1*_,*v*_*2*_,*…*, *v*_na_ are n_a_ weights of fuzzy rules, ${\overset{-}{\mu }}_{1},\;{\overset{-}{\mu }}_{2},\;\ldots ,{\overset{-}{\mu }}_{na}$ are the membership function values of each rule in FJWNN modeling, and PH is the prediction horizon.

In this section, the new fuzzy wavelet neural network model intended for system identification is introduced. The overall schematic diagram of the proposed FJWNN structure is shown in Fig 1. The structure is based on sub-jump wavelet neural network (sub-JWNN), fuzzy inference, and rule induction.

First, we focus on the sub-JWNN and its structure, as depicted in Fig 1(A). The sub-JWNNs, linear combinations of input regressors and wavelet neurons reside in the consequent parts of fuzzy rules in the proposed FJWNN model. The wavelet neurons include wavelets most effective on the output selected from a lattice of wavelets. The lattice of wavelets consists of different wavelets generated from a mother wavelet whose scale and shift parameters change at intervals [42]. In this study, the single-scale multi-dimensional Mexican hat wavelet is used as the mother wavelet:

$$\varphi \left(U\right)=\left(m-{∥U∥}^{2}\right)\text{exp}\left(-{∥U∥}^{2}/2\right)$$ (11)

where *U* and m are the input regressor vector and its dimension. The scaled and shifted variant of the Mexican hat wavelet was obtained using the following equation:

$$\varphi_{a_{i},\;B_{i}}\left(U\right)=\varphi \left(2^{-\;\left(a_{i}\;m/2\right)}\left(2^{a_{i}}U-B_{i}\right)\right)$$ (12)

where *a* is the scale parameter, *B* is the shift parameter vector. In the simulations of this paper, the values of scale parameters of the lattice of wavelets ranged from −4 to 4.

The main effective wavelets have the greatest impact on system modeling and hence are chosen from the wavelet lattice while in line with input linear regressors. This selection is initially made through the orthogonal least squares (OLS) method multiple times. Each time, a different number of main effective wavelets are selected, and the wavelets are combined with linear input regressors to model the training data. After choosing the best number of wavelets that minimized the root mean square error (RMSE) of the validation data, the genetic algorithm (GA) is used to search for different main effective wavelets. The best number is selected by replacing the initially selected wavelets with various wavelets from the wavelet lattice based on linear regressors, checking the validation RMSE, and choosing the best ones. The initial wavelets in the GA are main effective wavelets selected by OLS in the initial step. In our experiments, other GA parameters are selected as (population size = 1000, Generation steps = 40 and tolerance = 1e-5).

Having chosen the most effective wavelets, the wavelets are classified with the same scale parameter. For example, if i^th^ group has n_i_ wavelets with the same scale parameter a_i_, the output of the sub-JWNN is calculated using the following equation:

$$y_{sub-JWNN}=\overset{n_{i}}{\underset{j=1}{\sum }}w_{j}\;\varphi_{a_{i},\;B_{i}}\left(U\right)\;+\overset{m}{\underset{j=1}{\sum }}w_{u_{j}}\;u_{j}$$ (13)

where *a*_*i*_ is the scale parameter, *B*_*i*_ = [*b*_*1i*_, *b*_*2i*_, *…*, *b*_*mi*_] is the vector of the shift parameters of the i^th^ dominant wavelet and *U* = [*u*_*1*_, *u*_*2*_, *…*, *u*_*m*_] is the input regressor vector. Hence, the sub-JWNN was made of a linear combination of input regressors with wavelets with the same scale parameter chosen from the selected dominant wavelets.

The proposed FJWNN model structure, which is based on the sub-JWNN, is depicted in Fig 1(B). The structure is composed of different layers which models the input-output relation. In the first layer, the inputs *u*_*1*_, *u*_*2*_, *…*, *u*_*m*_ are entered into the fuzzification layer. The fuzzification step includes *n*_*a*_ fuzzy rules (*R*_*l*_, *l = 1*, *…*, *n*_*a*_) to produce the final output model.

$$\begin{array}{l} R_{l}\;:\;\;IF\;u_{1}\;is\;A_{1}^{l}\;AND\;\;u_{2}\;is\;A_{2}^{l}\;\;AND\;\ldots \;AND\;\;u_{m}\;is\;A_{m}^{l}\\ \;THEN\;\;\eta_{l}\;\;=\;\;y_{sub-JWNN_{l}} \end{array}$$ (14)

where each fuzzy rule corresponds to a single-scale parameter sub-JWNN, *n*_*a*_ is the number of rules (equal to the number of unique scale parameters of the selected dominant wavelets), the AND operator is the multiplication, and $A_{j}^{l}$ are Gaussian fuzzy membership functions calculated as follows:

$$\;A_{j}^{l}=\text{exp}\left(-\frac{1}{2}{\left(\frac{u_{j}-\;\;mu_{lj}}{su_{lj}}\right)}^{2}\right)\;,\;\;\;l=1,\;2,\ldots ,n_{a}\;\;,\;\;\;j=1,\;2,\ldots ,n_{l}$$ (15)

where *mu*_*lj*_ and *su*_*lj*_ are mean and standard deviation of Gaussian fuzzy membership functions. The *l*^*th*^ sub-JWNN has *m*_*l*_ inputs, (*n*_*l*_ *+m*) nodes in the hidden layer, and one output (*η*_*l*_).

In this study, to simplify the proposed FJWNN model and reduce the number of its parameters, fuzzy rule induction is applied through the imperialist competitive algorithm (ICA) [43]. Fuzzy rule induction consists of optimizing the structures of the antecedent part of fuzzy rules and allocating a weight for each rule to differentiate between fuzzy rules of different significance.

The antecedent part of a fuzzy rule includes an input membership function per input. Each input has *n*_*a*_ membership functions. In this study, the optimization of the structure of the antecedent part of fuzzy rules means firstly doubting the role of all inputs in the antecedent part of all fuzzy rules, and secondly choosing the optimal membership function among the *n*_*a*_ possible membership functions for an effective input in the antecedent part of each fuzzy rule. In the fuzzy rule induction procedure, for each fuzzy rule, there is an input vector and a corresponding antecedent vector, which specifies how the input vector participates in any of the rules. The antecedent vector members {*ca*_*i*_} can be 0, 1, …, *n*_*a*_. The zero value implies that the corresponding input does not play any role in that rule antecedent part, and nonzero numbers refer to the corresponding input membership function.

In (6), to determine the firing strength of the *l*^*th*^ fuzzy rule, the geometric mean of the membership functions of input variables which contribute in each rule antecedent is calculated, instead of just multiplying the functions in common neuro-fuzzy models.

$$\mu_{l}=\;\sqrt[{d_{l}}]{{\left(A_{1}^{l}\right)}^{c_{1}^{l}}\times {\left(A_{2}^{l}\right)}^{c_{2}^{l}}\times \ldots \times {\left(A_{m}^{l}\right)}^{c_{m}^{l}}}$$ (16)

where $d_{l}\;=\;\sum_{i\;=\;1}^{m}c_{i}^{l}$ and $c_{i}^{l}(l\;=\;1,\;2,\ldots ,n_{a}\;,\;i\;=\;1,\;2,\ldots ,m)$ are antecedent assignments represented as 0 or 1. The antecedent assignments are calculated as follows:

$$c_{i}^{l}=\text{sign}(ca_{i}^{l})$$ (17)

For weight assignment, a continuous weight *v*_*i*_ (*i* = 1, 2, …, *n*_*a*_) ranging from 0 to 1 is allocated to each of *n*_*a*_ fuzzy rules. This weight sets the significance of the rule in the proposed FJWNN model. Then, fuzzy rules with weights of smaller than a threshold are eliminated from the FJWNN model. Next, the defuzzification step is implemented, and the final output is calculated using the following equation:

$${\hat{y}}_{FJWNN}\left(k+PH\right)=\overset{r}{\underset{l=1}{\sum }}v_{l}\bar{\mu_{l}}\eta_{l}$$ (18)

where *PH* is the prediction horizon and

$$\bar{\mu_{l}}=\frac{\mu_{l}}{\sum_{j=1}^{n}\;\mu_{j}}$$ (19)

The unknown parameters of the FJWNN model include the mean and standard deviation parameters of Gaussian fuzzy membership functions and the weights of fuzzy rules adjusted using ICA and the weights of sub-JWNNs learned by the LS method [Table 1]. It should be noted that the scale and shift parameters of the dominant wavelets extracted by the OLS and the GA methods are fixed by ICA in the training phase. The dominant wavelets are selected from the wavelet lattice in the initial steps by the OLS and GA methods.

**Table 1:** Different methods used to train the unknown parameters of the proposed FJWNN model.

Unknown variables		Training method
1	Means and standard deviations of Gaussian fuzzy membership functions	ICA
2	Weights of fuzzy rules	ICA
3	Weights of sub-JWNN	LS

ICA is a computational method that is used to solve optimization problems. This method does not need the gradient of the cost function in its optimization process [43]. In our experiments, ICA parameters are selected as (Maximum Number of Iterations = 60, Population Size = 500, Number of Empires/Imperialists = 5, Selection Pressure = 1, Assimilation Coefficient = 1.5, Revolution Probability = 0.5, Revolution Rate = 0.3, Colonies Mean Cost Coefficient = 0.2).

**Fig 2:**
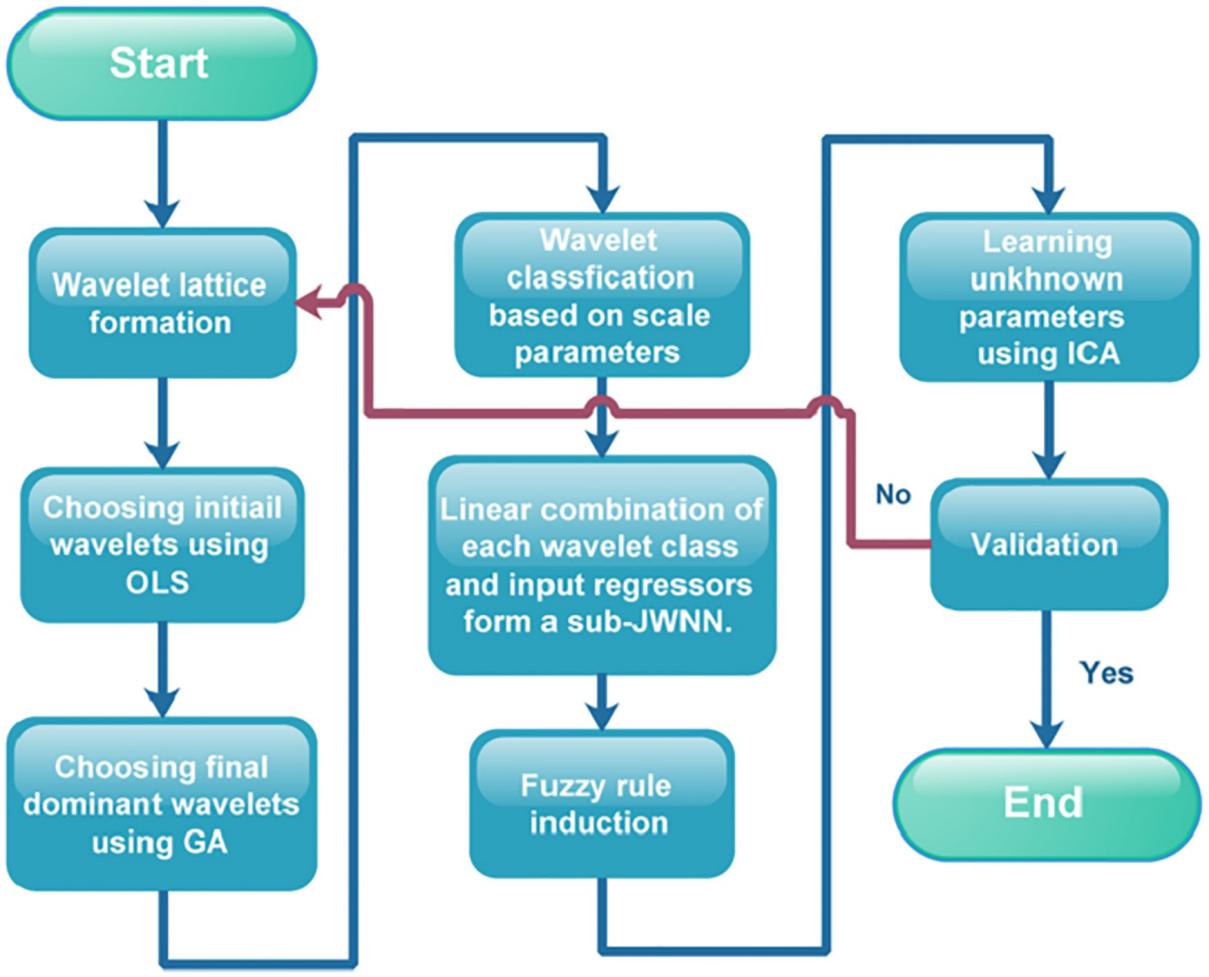
The proposed FJWNN modeling flowchart.

The flowchart of the proposed FJWNN modeling steps is presented in Fig 2.

### Performance metrics

The performance of the FJWNN model was evaluated in terms of the goodness-of-fit. Since the results of the multiple examples are compared with those of previous studies based on different performance metrics, the following performance metrics are first introduced: RMSE, root relative square error (RRSE), relative error (rel ERR%) and variance accounted for (VAF%).

The RMSE between the predicted (*y* hat) and measured output (*y*) is calculated using the following equation:

$$RMSE=\;\sqrt{\frac{1}{n}\overset{n}{\underset{i=1}{\sum }}{\left(y_{i}-{\hat{y}}_{i}\right)}^{2}}$$ (20)

In addition to RMSE, three fair performance indexes were used (RRSE, Rel ERR%, and VAF %). RRSE provides the RMSE of the predictions relative to the standard deviation of the measured output, and is obtained as follows:

$$\;RRSE=\sqrt{\;\left[\overset{n}{\underset{i=1}{\sum }}{\left(y_{i}-{\hat{y}}_{i}\right)}^{2}\right]/\left[\overset{n}{\underset{i=1}{\sum }}{\left(y_{i}-\bar{y}\right)}^{2}\right]}$$ (21)

The Rel ERR% provides the RMSE of the predictions relative to the mean square of the measured output, and is calculated using the following equation:

$$\;Rel\;ERR\;\%=\;100\;\times \;\sqrt{\;\left[\overset{n}{\underset{i=1}{\sum }}{\left(y_{i}-{\hat{y}}_{i}\right)}^{2}\right]\;/\;\left[\overset{n}{\underset{i=1}{\sum }}{\left(y_{i}\right)}^{2}\right]}$$ (22)

The VAF% provides the RMSE of the predictions relative to the mean square of the measured output, and is defined as follows:

$$VAF\%\;=\;100\;\times \;\left(1-\text{var}\left(y-\hat{y}\right)/\text{var}\left(y\right)\right)$$ (23)

## Results and discussion

In this section, the effectiveness of the proposed FJWNN model is evaluated using simulated and experimental examples. In examples 1–3, for each data set, ten independent runs are performed, and the mean and standard deviations (std) values of the accuracy metrics are calculated for both training and testing data. Correspondingly because of using rule induction method, for the number of rules (NOR) and parameters (NOP), in each case mean and std values are reported.

In this research, initial parameters of fuzzy membership functions are chosen, so that mean initial values are chosen randomly, and standard deviation initial values are chosen as 0.2. Initial fuzzy rules weights are set 0.7 and all initial antecedent parameters are set 1.

For our evaluation, we used a PC with Intel(R) Core(TM) i7-4700MQ CPU @ (2.40 GHz) and 8 GB RAM. All the methods were realized by MATLAB 7.12.

### Example 1—Function approximation

In this example, the proposed FJWNN model is evaluated on the piecewise single variable function formulated as (1). Moreover, the comparison between the present results and those obtained using the state-of-the-art models is provided in Table 2.

**Table 2:** The performance of different approximation methods in Example 1.

Method		NOR	NOP	Epoch	RRSE
**1**	**The proposed FJWNN**	6	45±5 (mean±std)	100	0.00010±0.00006 (mean±std)
**2**	**T2FWNN** [44]	4	24	200	0.01000
**3**	**FWN** [45]	--	27	300	0.00228
**4**	**FWNN**_**II**_ [46]	3	--	--	0.00140
**5**	**T2WNN** [5]	4	--	--	0.00060
**6**	**T2FWNN (multi-input)** [44]	4	84	200	0.00052
**7**	**FWNN** [47]	4	20	200	0.00044

NOR, Number of rules; NOP, Number of model parameters; RRSE, Root relative square error; FJWNN, Fuzzy jump wavelet neural network; 2FWNN, Type 2 fuzzy wavelet neural network; FWNN_II_, Fuzzy wavelet neural network II; T2WNN, Type 2 wavelet neural network; FWNN, Fuzzy wavelet neural network; -- No information is mentioned in the reference.

**Fig 3:**
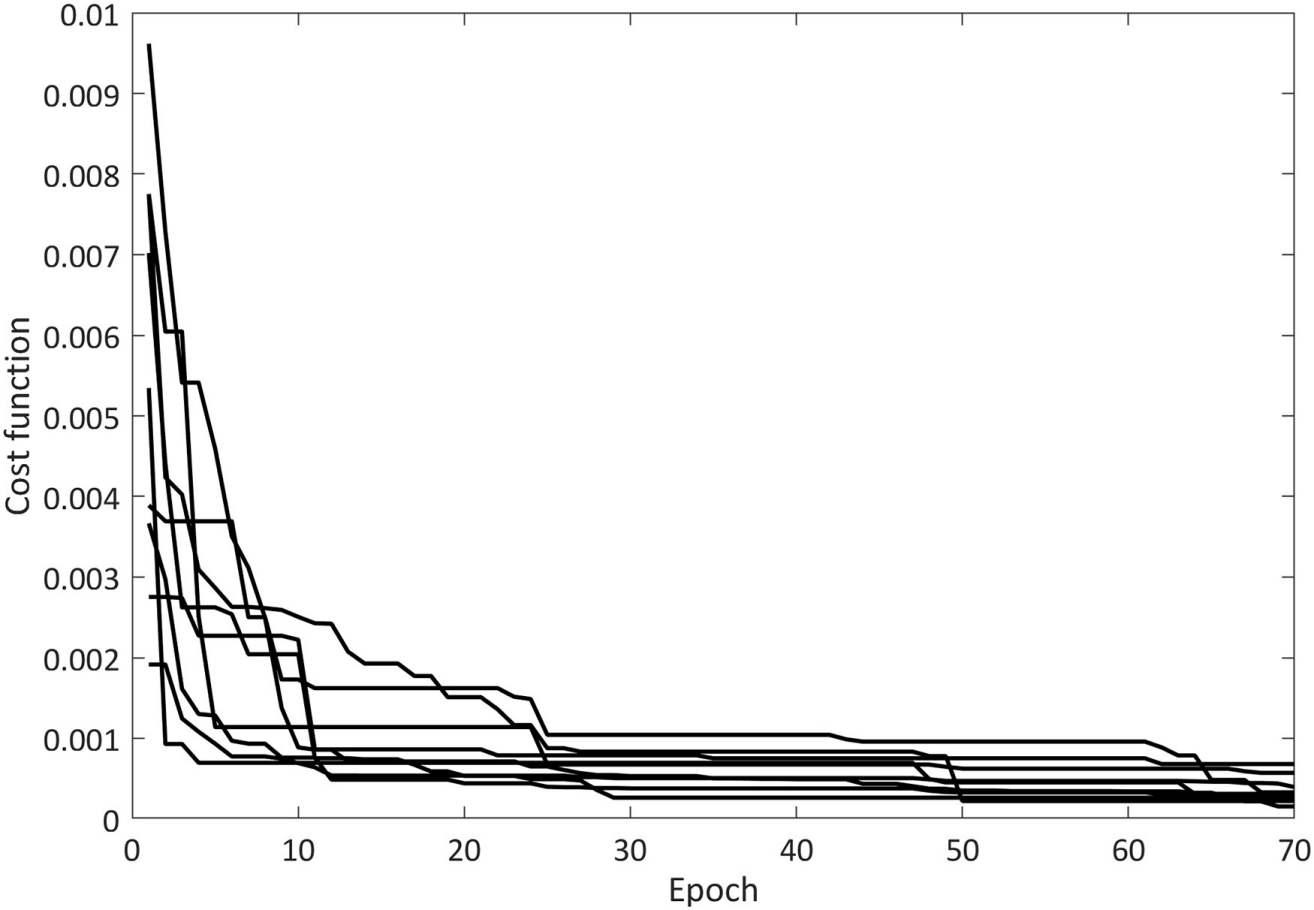
Cost function trend in ICA training procedure of FJWNN model for Example1 for ten independent runs.

According to the results, the proposed FJWNN model showed better performance compared to other models. Fig 3 shows how the cost function value reduced during epochs in the training procedure.

**Fig 4:**
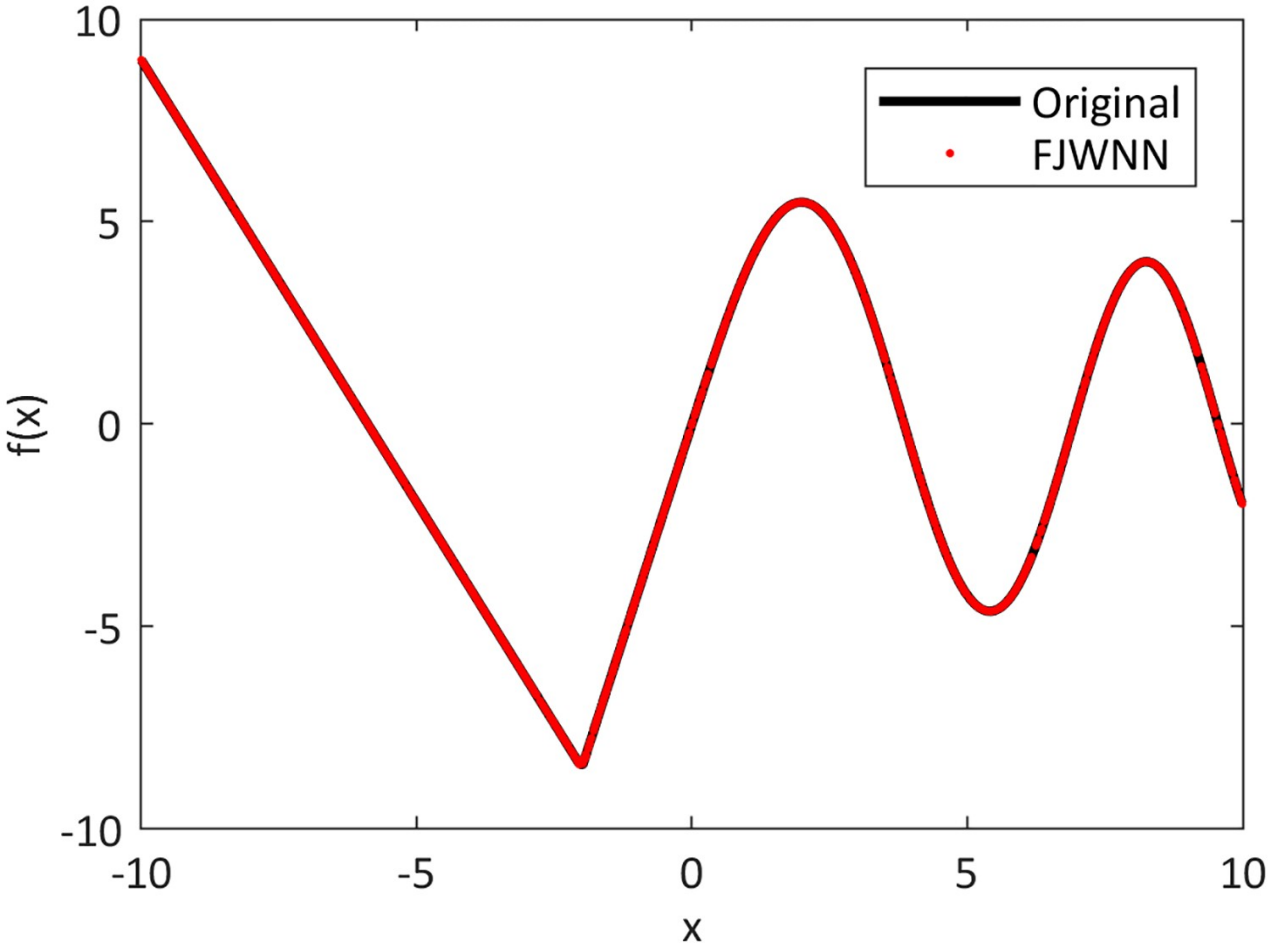
Comparison between the original piecewise single variable function and its FJWNN model output for the test data for ten independent runs.

The original piecewise single variable function and the FJWNN model output for the test data are depicted in Fig 4.

### Example 2—Dynamic nonlinear system identification

For dynamic nonlinear system identification, in the present study, five plants with different nonlinearity structure described in (2)–(6) are considered. For example, 2–1, in Table 3, the RMSE value of its FJWNN modeling is presented along with the corresponding values reported for recent models with the excitation signal as that in the present study. As can be seen in this table, the RMSE value of the FJWNN model is lower than those of other models.

**Table 3:** Comparison between the simulation results of different methods, for Example 2–1.

Method		NOR	NOP	Training RMSE	Test RMSE
**1**	**FJWNN**	3	41±2 (mean±std)	0.000023±0.000014 (mean±std)	0.000026±0.000026 (mean±std)
**2**	**FWNN** [4]	3	27	0.0197	0.0226
**3**	**FWNN** [4]	3	43	0.0187	0.0202
**4**	**PRWNN** [48]	--	48	--	0.0102
**5**	**Type-2 FWNN with FCM** [44]	4	33	0.0167	0.0187
**6**	**FWNN**[11]	2	30	0.0067	0.0163

NOR, Number of rules; NOP, Number of model parameters; RMSE, Root mean square error; FWNN, Fuzzy wavelet neural network; PRWNN, Pipeline recurrent wavelet neural network; FCM, Fuzzy C-means clustering; -- No information is mentioned in the reference.

**Fig 5:**
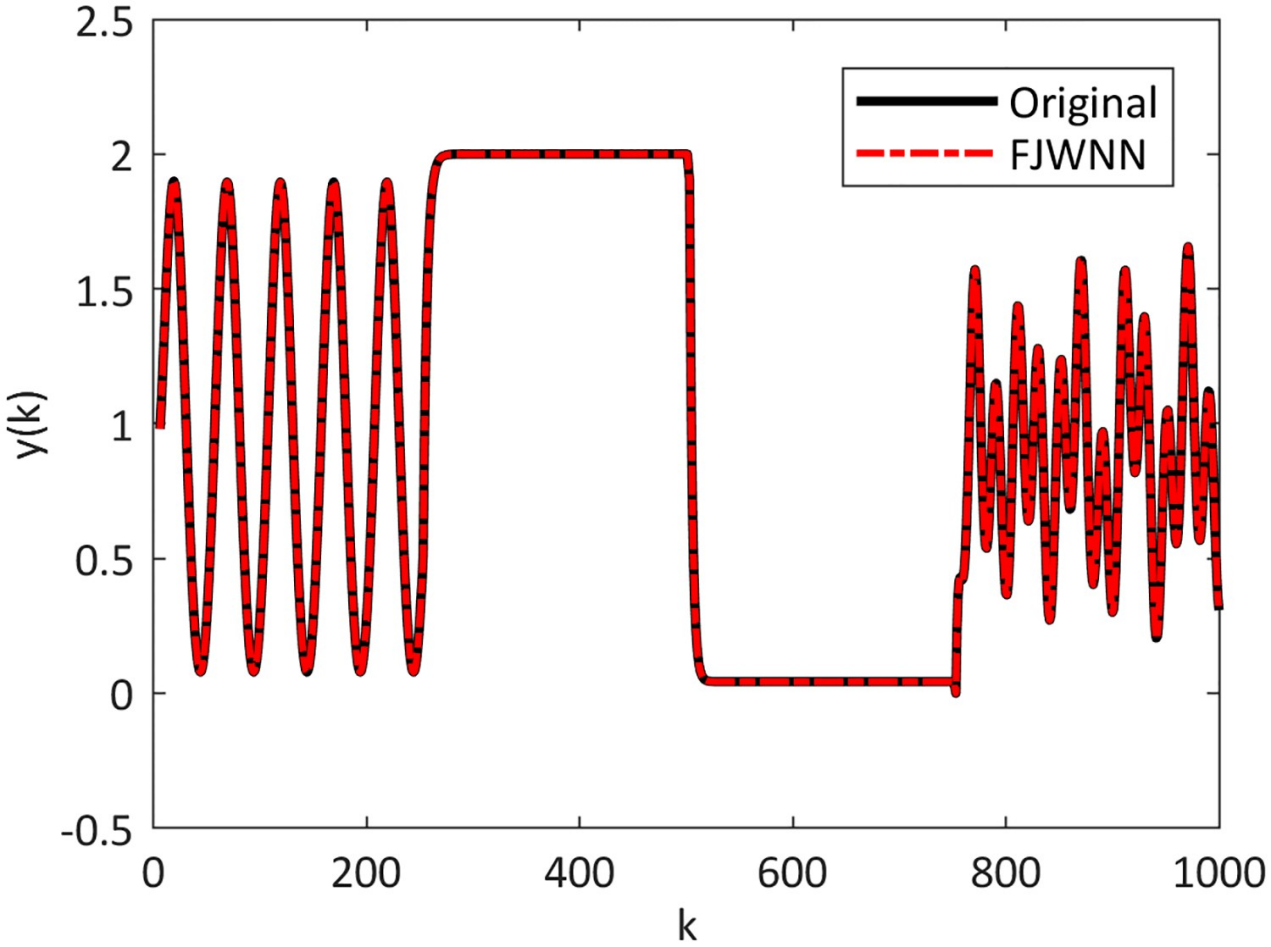
Comparison between the dynamic nonlinear system Example 2–1 and its FJWNN model estimation for the test data for ten independent runs.

**Fig 6:**
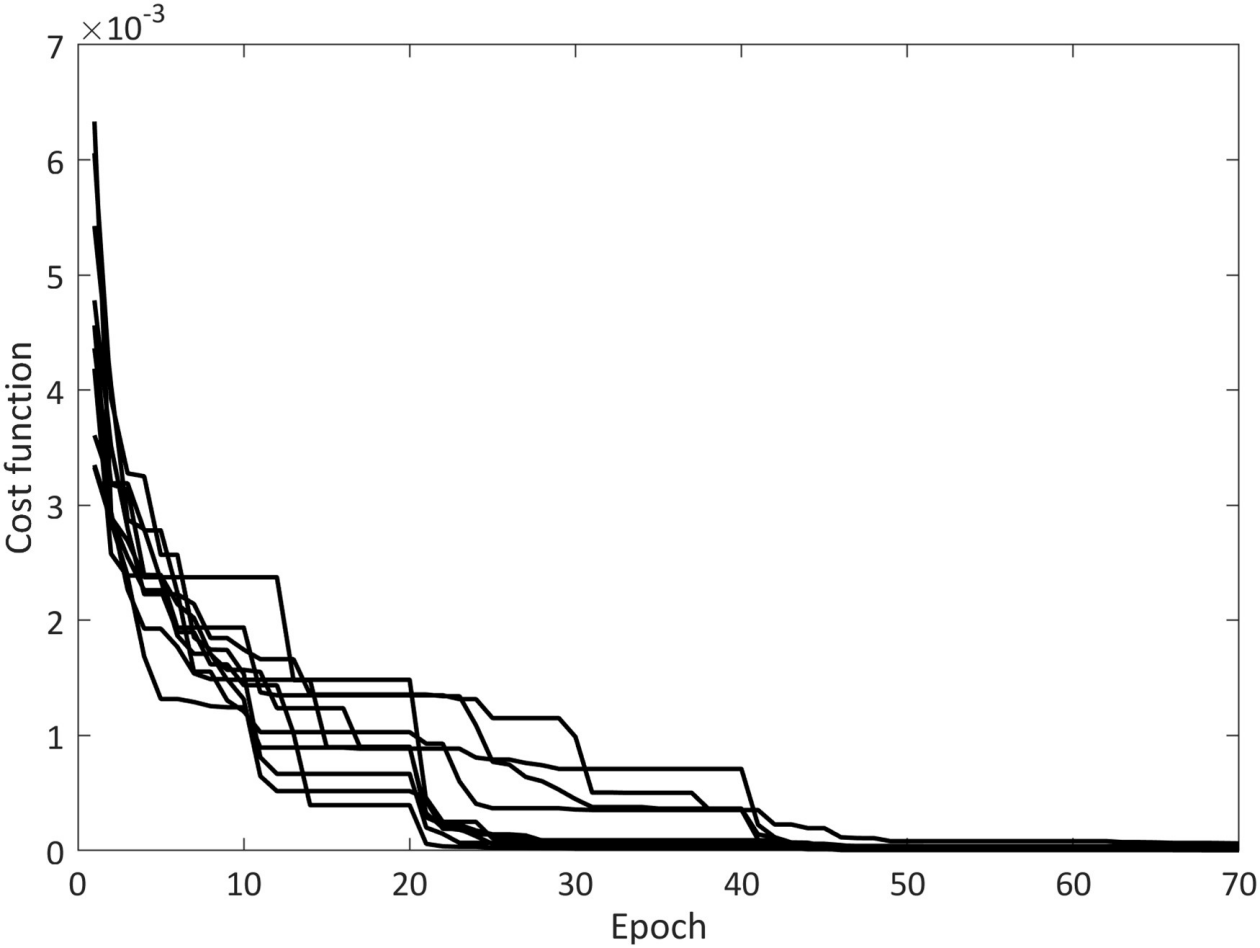
The cost function during ICA training of the proposed FJWNN model for Example 2–1 for ten independent runs.

Fig 5 shows the dynamic nonlinear system output and the output of the FJWNN model with three rules. Also, the cost function during the ICA training of the proposed FJWNN model is presented in Fig 6.

According to Table 3, the proposed FJWNN produced more acceptable results than other analyzed methods.

In the following, modeling of the other four nonlinear dynamics (examples 2–2 up to 2–5) are considered as system identification problem. In each case, in the training phase, a uniformly distributed random 1000 time steps signal over the interval [–1,1] is applied to the plant. While in [25], the training procedure continued for 50000 iterations during training phase. The comparison with other research works is summarized in Table 4.

**Table 4:** Comparison between the simulation results of different methods for Examples 2–2 up to 2–5.

RRSE of different Methods for test data		Example 2–2	Example 2–3	Example 2–4	Example 2–5
**1**	**FJWNN**	0.00020±0.00003 (mean±std)	0.0013±0.0010 (mean±std)	0.0114±0.0078 (mean±std)	0.0040±0.0002 (mean±std)
**2**	**PFLARNN** [27]	0.0453	0.0238	0.0198	0.0331
**3**	**FLARNN** [26]	0.0624	0.0379	0.0436	0.0428
**4**	**MLP** [24]	0.0773	0.1178	0.1607	0.0950

**Fig 7:**
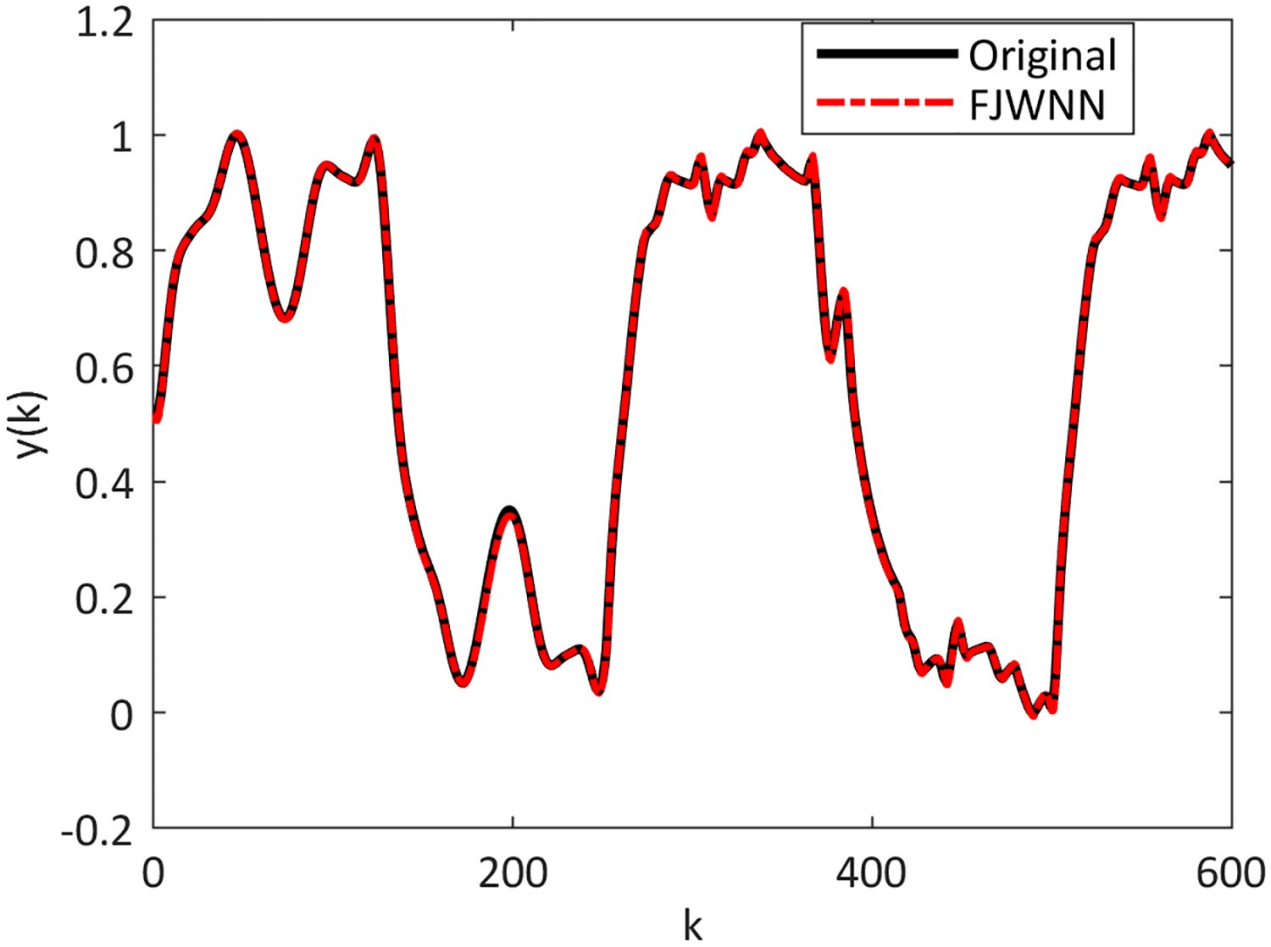
Comparison between the Example 2–2 and its FJWNN model estimation for test data for ten independent runs.

RRSE, Root relative square error; FWNN, Fuzzy wavelet neural network; PFLARNN, Pipelined functional link artificial neural network; PLARNN, Functional link artificial neural network; CFLANN, Chebyshev functional link artificial neural networks; MLP, Multiple layer perceptron;

The dynamic nonlinear examples and their FJWNN predictions for the test data are illustrated in Figs 7 up to 10.

**Fig 8:**
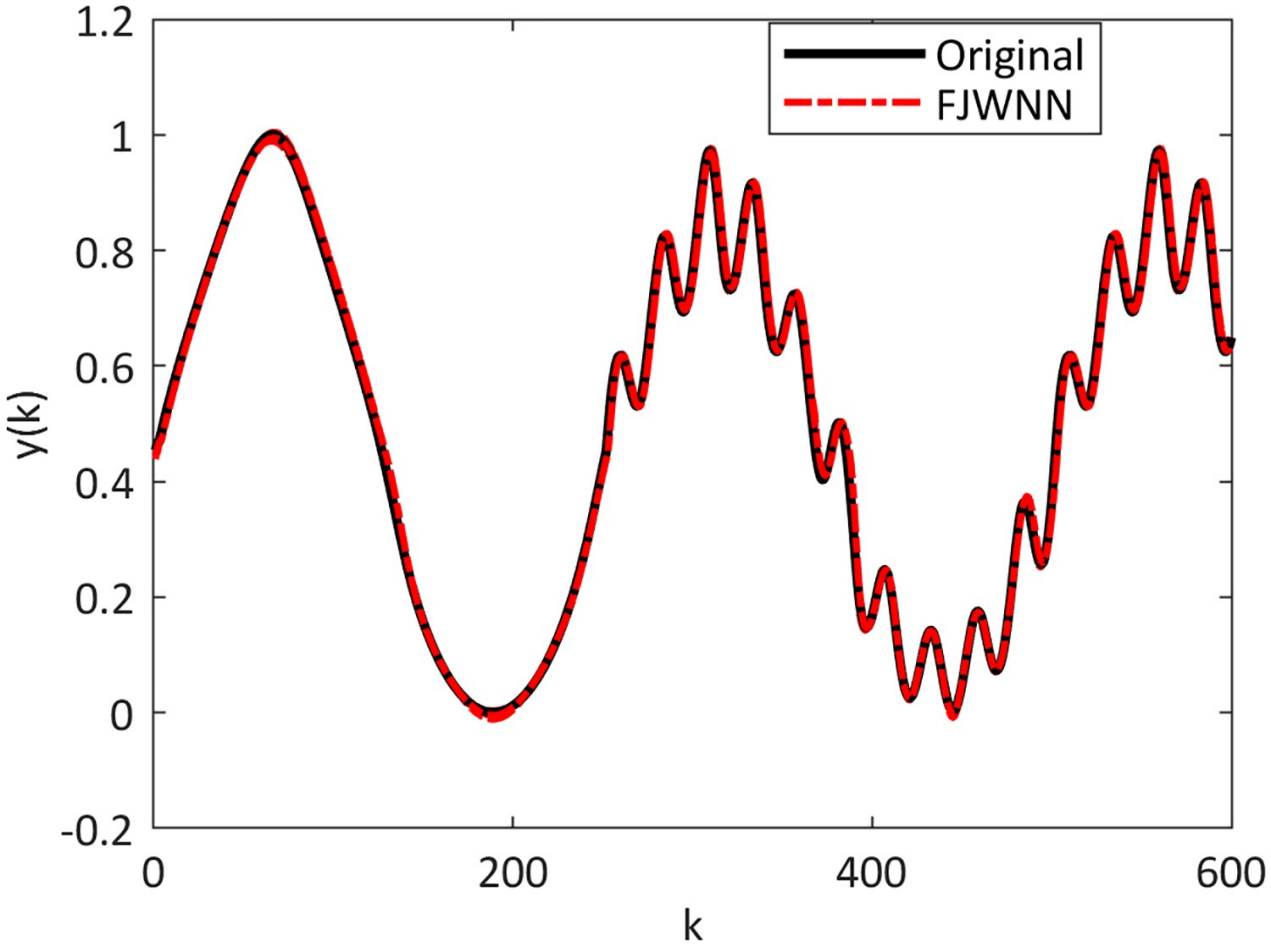
Comparison between the Example 2–3 and its FJWNN model estimation for test data for ten independent runs.

**Fig 9:**
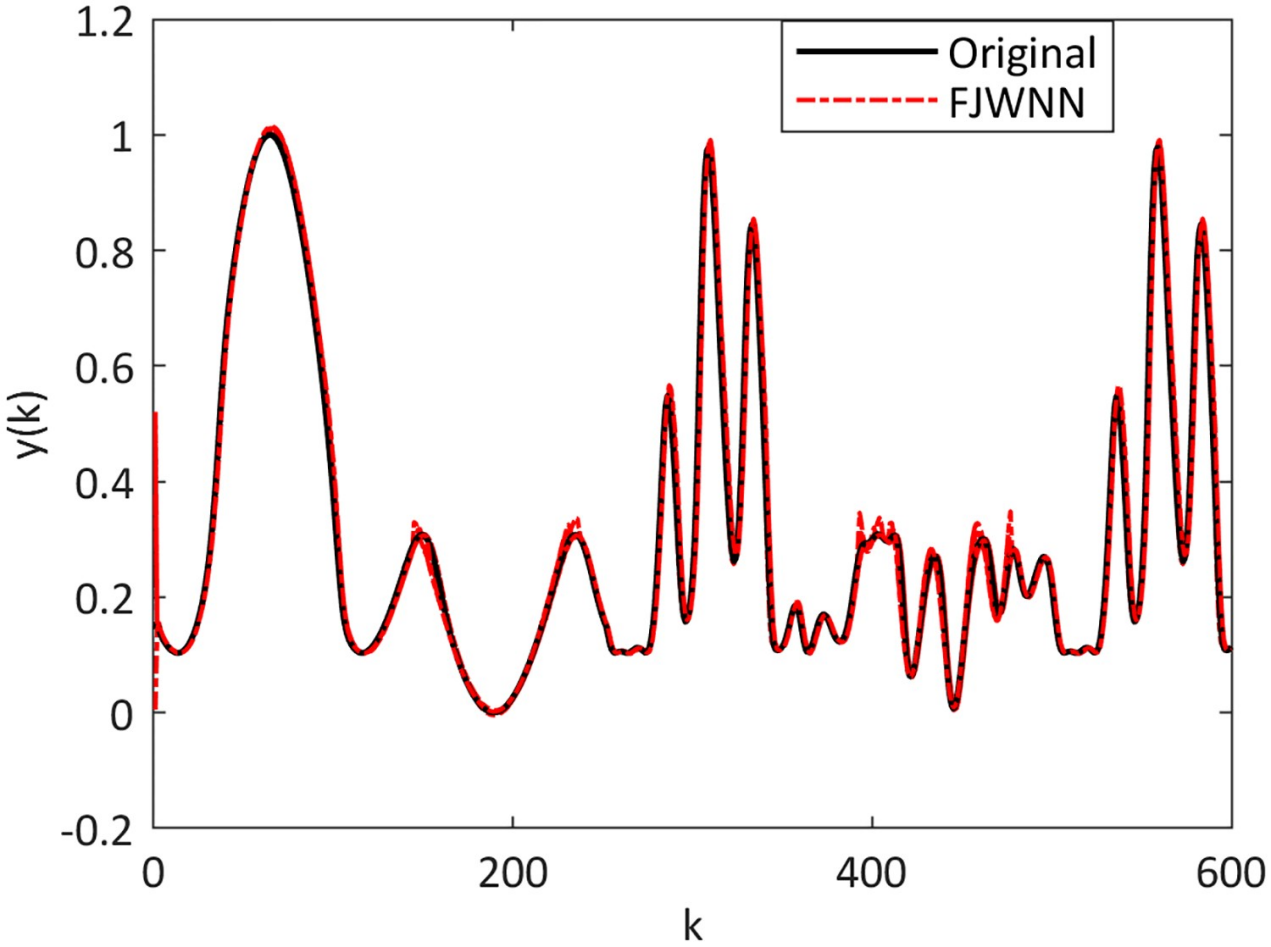
Comparison between the Example 2–4 and its FJWNN model estimation for test data for ten independent runs.

**Fig 10:**
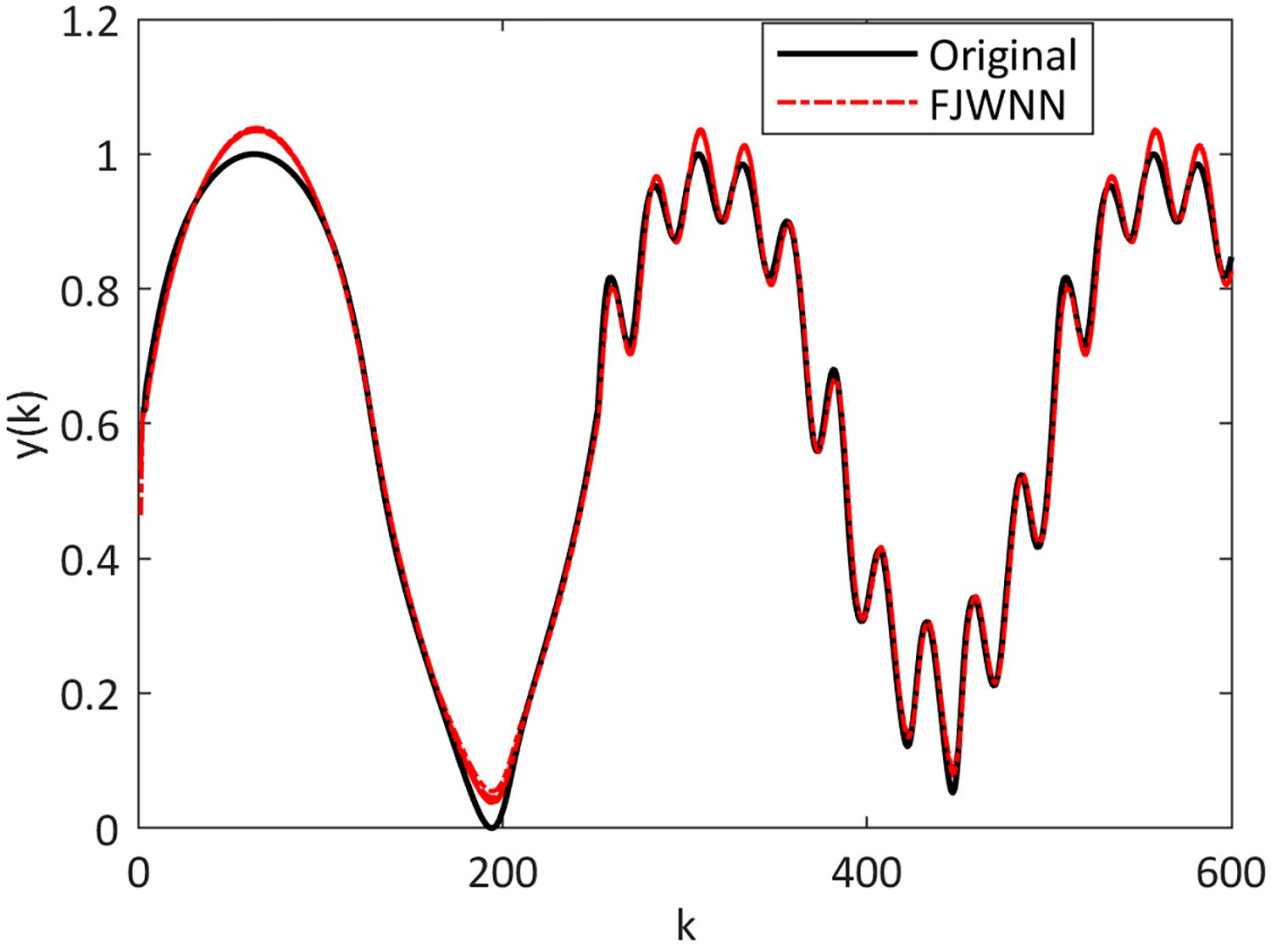
Comparison between the Example 2–5 and its FJWNN model estimation for test data for ten independent runs.

Simulation results for nonlinear dynamic identification systems described in (2)–(6) show that the proposed FJWNN leads to acceptable accuracy based on RMSE and RRSE metrics.

### Example 3—Predicting chaotic time series

**Fig 11:**
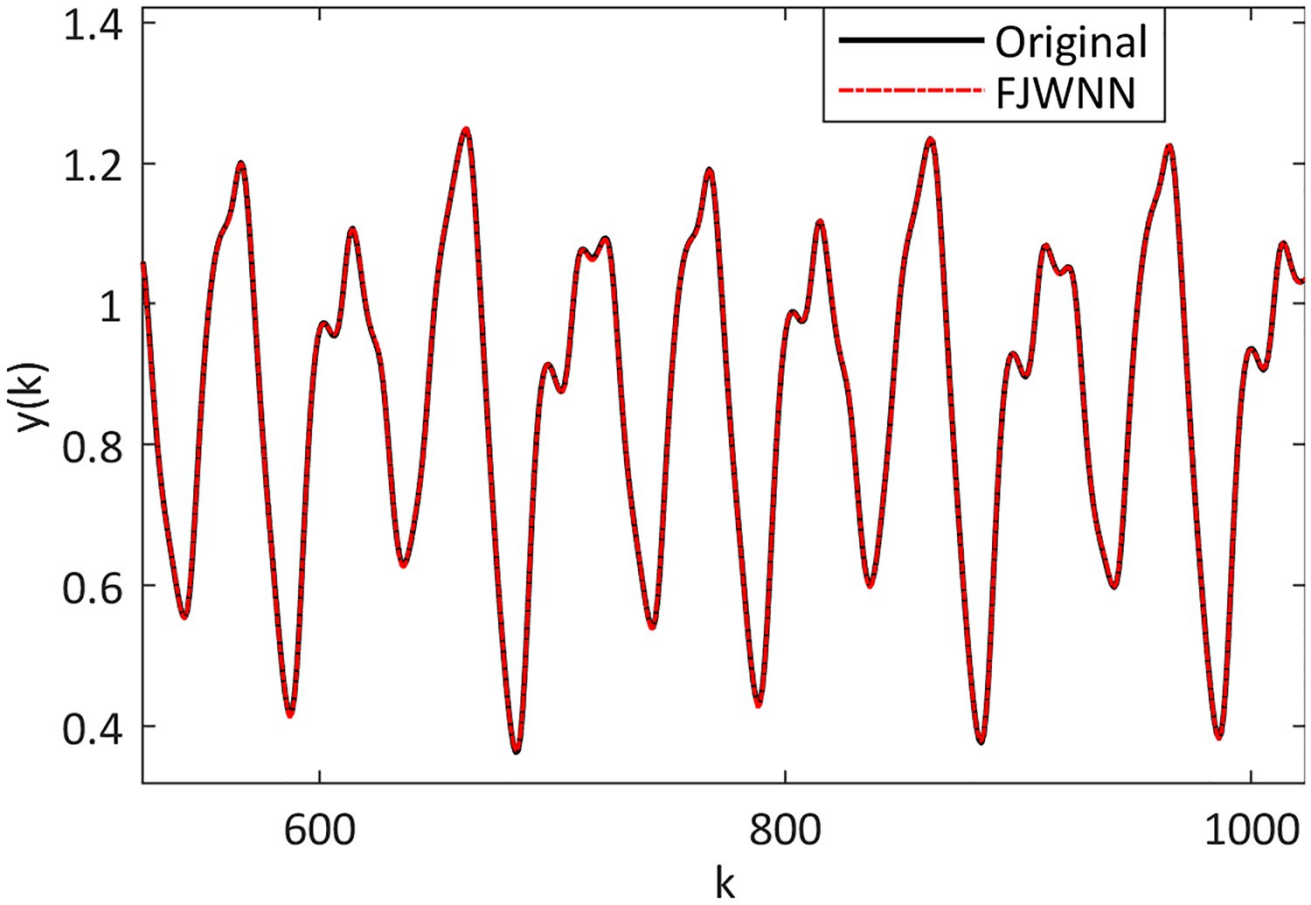
Comparison between the Mackey-Glass time series and its FJWNN prediction for the test data for ten independent runs.

**Fig 12:**
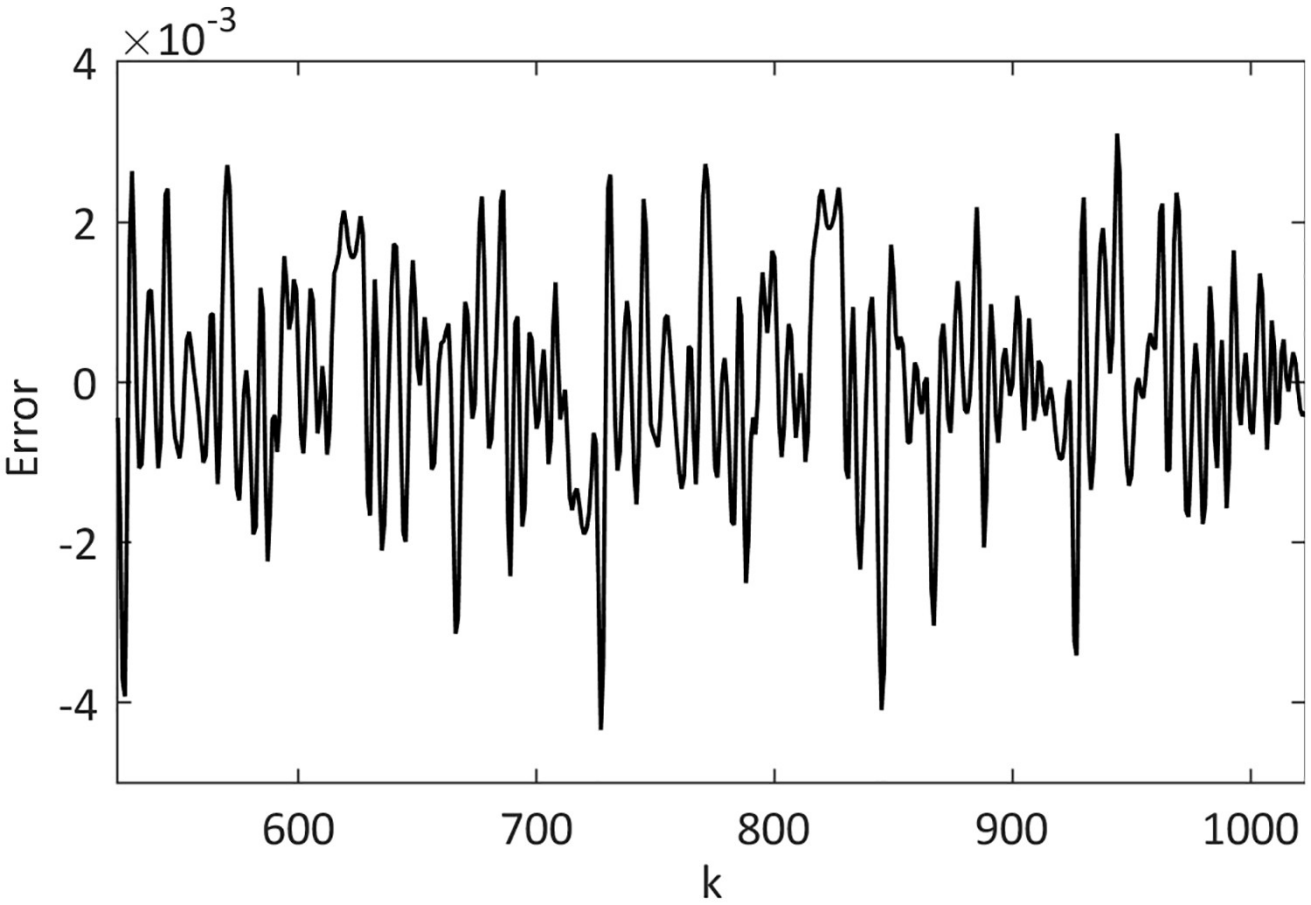
Mean of the test data prediction error of the proposed FJWNN model for Example 3 for ten independent runs.

This example dealt with the prediction of the chaotic Mackey-Glass time series to compare the prediction ability of the proposed FJWNN model with those of previous models in the presence of different SNR. The Mackey-Glass time series and its FJWNN prediction for the test data are illustrated in Fig 11, and the prediction error is shown in Fig 12.

The results of the comparison between the present model and some recent fuzzy neural and wavelet models are presented in Table 5. This table shows that the RMSE obtained by the FJWNN prediction is smaller than those obtained using the state-of-the-art models.

**Table 5:** Comparison of different methods results for Example 3.

Method		NOR	NOP	Epoch	Training RMSE	Test RMSE
**1**	**FJWNN**	5±1 (mean±std)	78±13 (mean±std)	100	0.00164±0.00044 (mean±std)	0.00159±0.00042 (mean±std)
**2**	**ASOA-FNN** [49]	6	--	--	0.00910	0.00540
**3**	**IT2FNN-3** [28]	16	--	50	--	0.00200
**4**	**AWN (first order)** [31]	--	96	4000	0.00183	0.00178
**5**	**Locally liner neural fuzzy** [33]	--	47 neurons	47	--	0.00788*
**6**	**FWNN** [47]	3	51	70	--	0.00300

NOR, Number of rules; NOP, Number of model parameters; RMSE, Root mean square error; FJWNN, Fuzzy jump wavelet neural network; AESN, Adaptive echo state network; IT2FNN, Interval type-2 fuzzy neural networks; ASOA-FNN, Adaptive second-order algorithm-based fuzzy-neural-network; FWNN, Fuzzy wavelet neural network; DOS-FCM, Distance orientation similarity-based fuzzy C-means algorithm; -- No information is mentioned in the reference;

* The prediction error in its reference was described in RRSE metric.

Chaotic degree (τ) from Mackey–Glass series is used as an uncertainty source and variated to compare the performance of the proposed FJWNN model with state-of-the-art models. Corresponding results are reported in Table 6. It shows FJWNN architecture performs better than ANFIS and IT2FNN when uncertainty degree is increased.

**Table 6:** The noise-free Mackey–Glass chaotic time-series prediction (τ = 13, 30, 100).

Model		RMSE for τ = 13	RMSE for τ = 30	RMSE for τ = 100
1	FJWNN	7.5140e-4±1.419e-4	0.0975±0.0059	0.1564±0.0165
2	ANFIS [30]	2.0196e-4	0.1792	0.4678
3	IT2FNN [28]	2.0014e-4	0.1165	0.2132

FJWNN, Fuzzy jump wavelet neural network; ANFIS, Adaptive neuro-fuzzy inference system; IT2FNN, Interval type-2 fuzzy neural networks, Root mean square error; τ, Chaotic degree;

In the next step, the Mackey-Glass time series are corrupted with noise levels of 0dB, 10 dB, and 20 dB of SNR (signal-to-noise ratio) as a high source of uncertainty. Table 7 shows that when different levels of noise are added, FJWNN model performs better than ANFIS and IT2FNN. This is because FJWNN model handles noise better because of choosing dominant wavelets in the proposed structure optimization procedure.

**Table 7:** Corrupted by uniformly-distributed stationary additive noise Mackey–Glass chaotic time series prediction (τ = 17).

Model		RMSE forSNR = 0	RMSE for SNR = 10	RMSE for SNR = 20
1	FJWNN	0.0904 ±0.0109	0.0415±0.0007	0.0153±0.0006
2	ANFIS [30]	0.2506	0.1031	0.3333
3	IT2FNN [28]	0.2125	0.0722	0.0143

τ, Chaotic degree; SNR, Signal noise ratio; FJWNN, Fuzzy jump wavelet neural network; ANFIS, Adaptive neuro-fuzzy inference system; IT2FNN, Interval type-2 fuzzy neural networks, Root mean square error;

Furthermore, the proposed FJWNN consists of fuzzy rules with different weights as their importance optimized in the training procedure to make fuzzy rule induction plausible. During the training procedure, the fuzzy rules with weights of smaller than a given threshold (0.05) were eliminated from the FJWNN model by setting their weights to zero, as for one of FJWNN models shown in Table 8.

**Table 8:** Different weights of the FJWNN model for Example 3.

Rule number	1	2	3	4	5
**Fuzzy rule weights**	0.0127	0.0276	0.0210	0.7790	0.7445
**Modified weights**	0	0	0	0.7790	0.7445

### Example 4—Real-world Box-Jenkins gas furnace system

In this real application, the proposed FJWNN model is evaluated on the gas furnace benchmark dataset. Moreover, the comparison between the present results and those obtained using the state-of-the-art models is provided in Table 9.

**Table 9:** Comparison of different methods results for Example 4.

Method		NOR	NOP	Epoch	Training RMSE	Test RMSE
**1**	**FJWNN**	2	25	100	0.1581	0.3421
**2**	**ANFIS-FCM** [25]	2	--	--	0.1600	0.4900
**3**	**FNN-GSA** [50]	3	--	--	0.1225	0.3834
**4**	**FNN-APTGA** [51]	2	--	--	0.1400	0.3700
**5**	**FBLS** [1]	4	--	--	0.1618	0.3479

**Fig 13:**
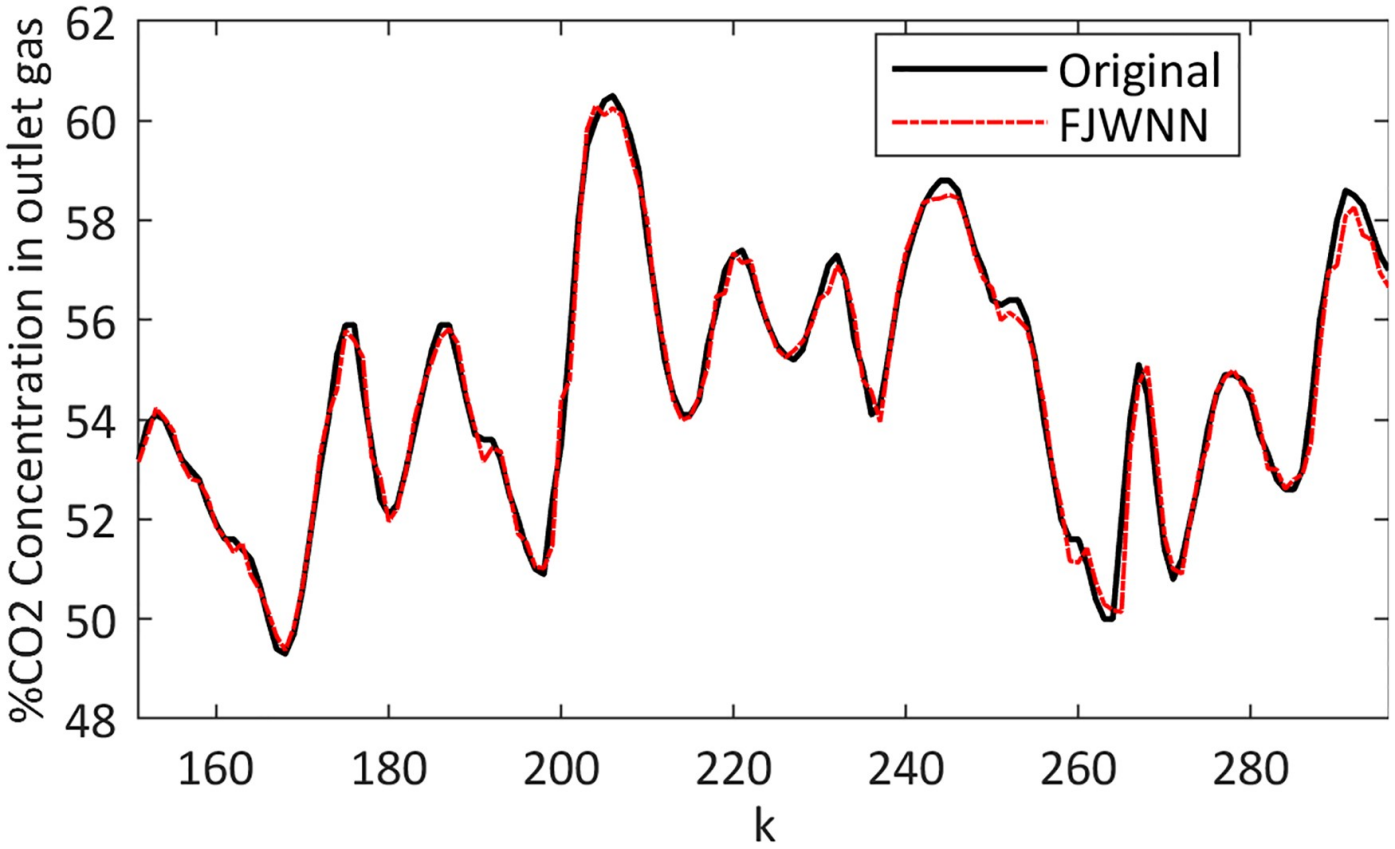
Comparison between the gas furnace output and its FJWNN prediction for the test data.

The gas furnace output and its FJWNN prediction for the test data are illustrated in Fig 13. To compare these results with existing models that have been applied to the same process, the root mean square error (RMSE) is used. The comparison results in Table 9 indicate that the FJWNN model can achieve higher accuracies.

### Example 5—EMG signal modeling problem

**Fig 14:**
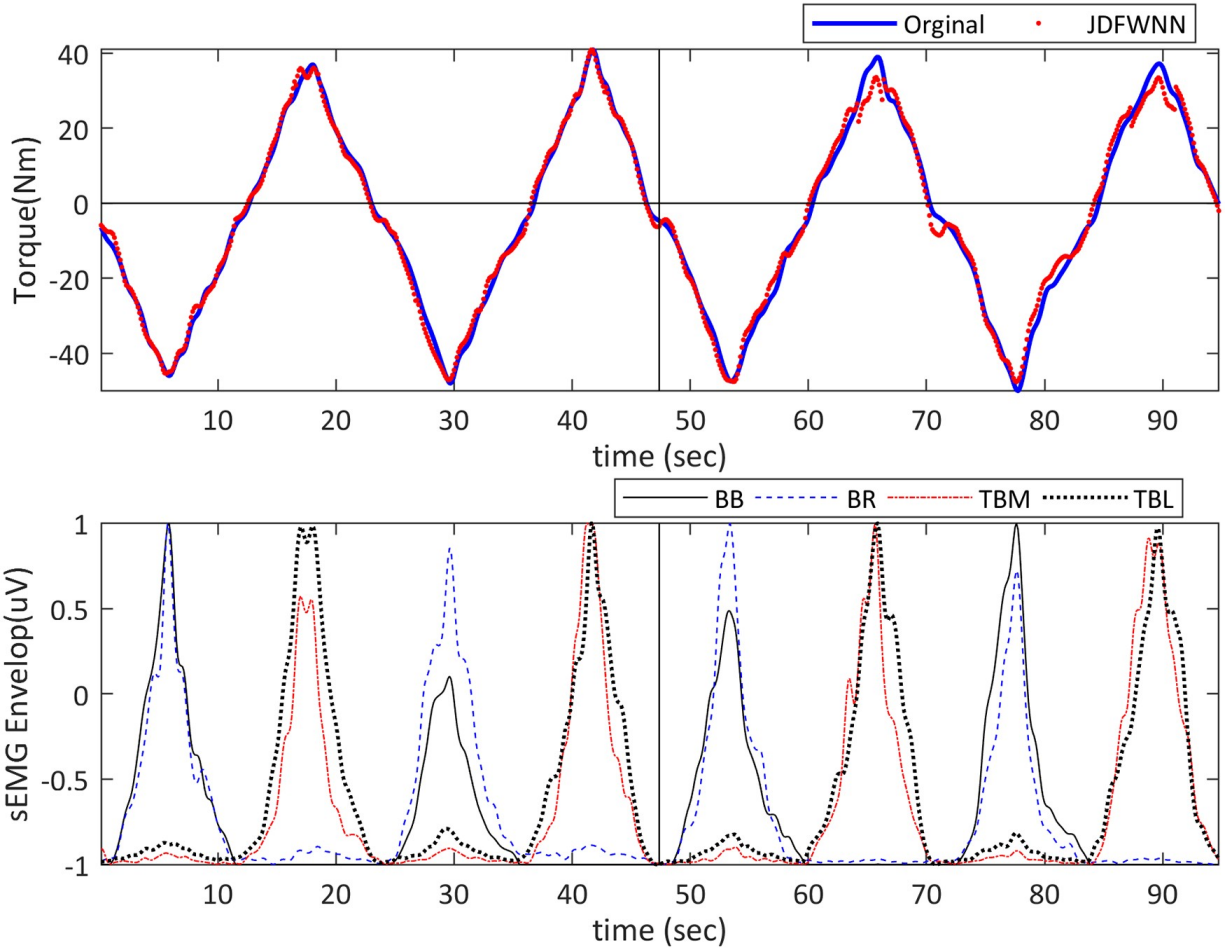
50% MVC elbow flexion-extension isometric ramps of the third participant. (A) recorded torque (solid blue) and estimated torque (dotted red) for each muscle (upper), and (B) the corresponding SD sEMG envelopes (bottom). The two sections of sEMG envelopes and recorded torque separated by thick black lines were used to train and test correspondingly.

In this example, the proposed FJWNN model was used to model EMG torques using experimental data. Fig 14. presents recorded and estimated torques and sEMG envelopes for the third participant. Considering this participant, an epoch of 47 sec was used for training, and the remaining time was used for testing the proposed FJWNN. As shown in Fig 14, the estimated torque signal followed the measured signal very well (Rel Err% = 7.13).

Table 10 provides the results of Rel Err% on SD records for 5 participants during elbow flexion-extension isometric ramps at 30%, 50%, and 70% MVC in comparison with the results obtained by [36].

**Table 10:** The rel ERR% results of FJWNN model estimation on SD records obtained through elbow flexion-extension isometric ramps (test dataset) for five participants at 30%, 50%, and 70% maximum voluntary contractions (MVC).

Rel Err %		S1	S2	S3	S4	S5
**30%**	**Ref** [36]	14.60	10.60	13.90	--	22.20
	**FJWNN**	**14.06**	**8.98**	**12.17**		**18.99**
**50%**	**Ref** [36]	12.30	10.30	11.10	11.40	24.00
	**FJWNN**	**11.70**	10.97	**10.25**	12.27	**15.80**
**70%**	**Ref** [36]	14.70	--	16.40	--	11.30
	**FJWNN**	**12.65**		**10.84**		**12.29**

Rel Err, Relative error; -- There were missing data in the dataset.

Table 11, on the other hand, presents the comparison between the VAF% results of the present study (in terms of mean±std) and those obtained in [38, 52].

**Table 11:** The VAF% (mean±std) results of FJWNN modeling on SD records obtained through elbow flexion-extension isometric ramps (test dataset) for all participants at 30%, 50%, and 70% MVCs.

MVC percentage		VAF%		
		mean±std	minimum	Maximum
**30%**	**Nonlinear dynamic model** [52]	80.86±13.12	65.92	92.82
	**Neuro-fuzzy model** [38]	95.58±2.85	91.38	97.55
	**FJWNN**	**97.73±1.33**	**96.20**	**99.18**
**50%**	**Nonlinear dynamic model** [52]	91.06±6.14	83.44	96.30
	**Neuro-fuzzy model** [38]	98.54±0.78	97.57	99.19
	**FJWNN**	98.39±0.59	97.44	98.92
**70%**	**Nonlinear dynamic model** [52]	89.74±5.16	86.08	96.4
	**Neuro-fuzzy model** [38]	94.64±5.37	88.50	98.48
	**FJWNN**	**98.61±0.22**	**98.41**	**98.83**

VAF, Variance accounted for; MVC, Maximum voluntary contractions; std, standard deviation; FJWNN, Fuzzy jump wavelet neural network; -- There were missing data in the dataset.

Furthermore, the cross-checking results of the FJWNN model torque estimation on SD and DD records for elbow flexion-extension at 30% and 70% MVCs are presented in Table 12. The cross-checking test included training the proposed model by records for elbow flexion-extension at 50% MVC, and testing the model by the records for elbow flexion-extension at 30% and 70% MVCs.

**Table 12:** Cross-checking Rel Err% results of FJWNN model estimation in comparison with the results of [36] on SD and DD records obtained through elbow flexion-extension isometric ramps for five participants at 50% MVC (for model training) and 30% and 70% MVCs (for model testing).

Test Rel Err%			S1	S2	S3	S5	mean±std
**SD**	**FJWNN**	**30% MVC**	**18.03**	**10.52**	**18.02**	**25.98**	**13.21±3.39**
		**70% MVC**	**16.41**	--	**17.80**	**17.70**	
	[36]	**30% MVC**	23.80	13.70	21.90	35.10	25.30±6.70
		**70% MVC**	28.10	--	26.10	28.40	
**DD**	**FJWNN**	**30% MVC**	**17.45**	**11.87**	18.45	**36.26**	**17.49±7.56**
		**70% MVC**	**22.40**	--	**24.54**	**17.66**	
	[36]	**30% MVC**	29.70	16.40	15.20	42.20	**28.70±10.00**
		**70% MVC**	37.00	--	30.90	29.50	

Rel Err, Variance accounted for; MVC, Maximum voluntary contractions; std, standard deviation; SD, single differential signal; DD, double differential signal; FJWNN, Fuzzy jump wavelet neural network; -- There were missing data in the dataset.

An overview of the number of cross-modeling parameters is presented in Table 13. For proposed FJWNN model, the number of fuzzy rules, number of antecedent parameters and total number of parameters with and without using fuzzy rule induction are compared. As shown, the effect of using fuzzy rule induction in reducing the number of model parameters is observed.

**Table 13:** The number of rules (NOR), the number of fuzzy rule antecedent parameters (NOA), and the number of parameters (NOP) for FJWNN modeling on SD records in the cross-checking.

Methods	S1			S2			S3			S5		
	NOR	NOA	NOP	NOR	NOA	NOP	NOR	NOA	NOP	NOR	NOA	NOP
**FJWNN with fuzzy rule induction**	5	26	41	6	30	46	5	26	41	3	14	27
**FJWNN without fuzzy rule induction**	5	40	55	6	48	64	5	40	55	3	24	37
TSK neuro-fuzzy model with four input, one output*	5	40	65	6	48	78	5	40	65	3	24	39

NOR, Number of rules; NOA, number of fuzzy antecedent parameters; NOP, number of parameters;

*, The proposed fuzzy linear TSK system has four inputs and one output and all of the input fuzzy membership functions are Gaussian; TSK, Takagi-Sugeno-Kang.

Fuzzy rule induction is also used to organize the antecedent parts of the fuzzy rules, reducing the number of parameters. For example, in Table 13, less unknown parameters are to be determined for FJWNN model for the antecedent parts of fuzzy rules. In the same case, if the generation of the Gaussian membership functions for a TSK neuro-fuzzy model with four input, one output, and an equal number of fuzzy rules is intended, then the number of model parameters is expected to be more as [38]. So the number of parameters is significantly decreased for the present FJWNN model proposed for EMG cross-modeling.

The proposed fuzzy model resulted in %VAF (mean ± std) = 98.24 ± 0.71 for all trial signals. The best performance of the model in [38] yielded the %VAF (mean ± std) of 96.40 ± 3.38. Thus, the proposed FJWNN model improved torque modeling results. Overall, there is an improvement in the reconstructed torque performance criterion of the proposed FJWNN, compared to those of the models in [36, 38, 52].

One of the limitations of the proposed FJWNN method is its running time during the learning procedure. For example, the average running time of its Matlab implementation for the examples 1, 2–1, 2–2, 2–3, 2–4, 2–5, 3, 4, and experimental EMG signals were 27±10, 77±12, 3±4, 23±1, 23±10, 1±1, 44±14, 4±1, and 11±5 (in minutes). This implementation, in its current form, is not thus suitable for online applications. The Vectorization packages with C++ implementation could be used to reduce the running time, which is the focus of our future work. The running time of the trained system on the test set is, however, acceptable. For example, the running time of analyzing the test set for the first example was 0.02±0.23 (in sec).

Now, a comparison of the performance of the proposed FJWNN with those of the state-of-the-art models was presented, evidencing the superior performance of the proposed model. For nonlinear systems, using models with fewer parameters lead to missing essential relationships in the data, while using models with many parameters makes parameter estimation difficult. However, in most cases, many model terms are redundant, and only a few significant terms with a specific accuracy are necessary. In the present study, the OLS and GA methods were used to select dominant wavelets which described nonlinear behavior, delivering a restricted number of wavelets in the sub-JWNN model. Accordingly, the number of wavelet classes decreased, and the structure of the proposed FJWNN model became noticeably simpler.

Moreover, choosing dominant wavelets using the OLS and GA methods can reduce the initial values of the cost function (See Figs 3 and 6). In the presented approach, fuzzy rule induction removes ineffective parameters or rules to simplify the proposed FJWNN model. Based on the overall analysis using the simulation and real data, it can be concluded that the proposed FJWNN model has high precision and less complexity, compared to the state-of-the-art models.

According to the performance of the FJWNN model for the real data in examples 4 and 5, the proposed FJWNN model employs the learning ability of neural networks, time-frequency localization property of wavelets and approximate reasoning characteristics of fuzzy systems to present the effective technique to deal with uncertainty and disturbances in real data for complex hybrid nonlinear-linear problems.

## Conclusions

In this paper, FJWNN combined with rule induction as a new wavelet-based identification model was proposed for the identification of real data dynamic nonlinear-linear systems. In the proposed approach, OLS and GA methods are respectively used to choose dominant wavelets along with the linear combination of input regressors. Fuzzy rules including wavelets with various scale parameters (different resolutions) can capture different behaviors (global or local) of the systems. Then, by applying fuzzy rule induction and assigning a weight to each fuzzy rule, which determines the importance of each fuzzy rule, insignificant rule is removed. Also fuzzy rule induction prunes unnecessary inputs from each of fuzzy rules. The obtained results of simulation and experimental examples demonstrate that the proposed model is quite useful in dynamic nonlinear system identification. Overall, the proposed FJWNN model can be considered a promising tool for EMG‑Torque modeling. Possible future work will be the utilization of the FJWNN model in on-line identification of dynamic nonlinear systems with real data applications.
